# Equitable Donor Assessment Model of Deceased Donor Kidney Quality

**DOI:** 10.1016/j.ekir.2026.103790

**Published:** 2026-01-21

**Authors:** Hatem Ali, Ahmed Daoud, Mahmoud Mohammed, Samar Abd ElHafeez, Miklos Z. Molnar, Jolanta Malyszko, Charat Thongprayoon, Wisit Cheungpasitporn, Tibor Fülöp

**Affiliations:** 1 Renal Department, University Hospitals of Wales, Cardiff, UK; 2 Divison of Nephrology, Department of Medicine, Medical University of South Carolina, Charleston, South Carolina, USA; 3 Medicine Services, Ralph H. Johnson VA Medical Center, Charleston, South Carolina, USA; 4 Division of Nephrology, Department of Medicine, Southwest Nephrology Associates, Chicago, Illinois, USA; 5 Epidemiology Department, High Institute of Public Health, Alexandria University, Alexandria, Egypt; 6 Division of Nephrology and Hypertension, Department of Internal Medicine, Spencer Fox Eccles School of Medicine at the University of Utah, Salt Lake City, Utah, USA; 7 Department of Nephrology, Medical University of Warsaw, Poland; 8 Division of Nephrology and Hypertension, Department of Medicine, Mayo Clinic, Rochester, Minnesota, USA

**Keywords:** donor-derived factors, equitable assessment, graft survival, kidney quality assessment, organ allocation, organ distribution fairness

## Abstract

**Introduction:**

The Kidney Donor Profile Index (KDPI) guides organ allocation but blends donor and recipient influences, potentially misclassifying organ quality and contributing to inequity. We developed the Equitable Donor Assessment Model (EDAM), a donor-focused index that isolates intrinsic graft-failure risk independent of recipient survival.

**Methods:**

Using the United Network for Organ Sharing (UNOS) data (2010–2020; *N* = 122,646), we modeled death-censored graft failure with death as a competing event using Fine-Gray regression. Donor coefficients were adjusted for recipient and transplant covariates—including human leukocyte antigen (HLA) mismatch and ischemia time—to derive donor-specific subhazard ratios (SHRs) for the EDAM score. Performance was evaluated using Harrell’s C-index and internal-external cross-validation across 5 geography-aligned US super-regions.

**Results:**

Higher donor age, diabetes, hypertension, stroke as cause of death, proteinuria, cytomegalovirus (CMV) seropositivity, and elevated creatinine independently increased graft-failure risk. EDAM demonstrated robust discrimination (C-index = 0.69) and excellent calibration across US regions (pooled slope = 1.02; intercept = −0.002). Stratification into 5 data-driven categories showed a graded rise in cumulative incidence of graft failure (Gray’s *P* < 0.05). Nearly half of kidneys classified as moderate-to-high KDPI (≥ 0.20) were reclassified as low-risk by EDAM (< 0.80) and achieved identical 10-year graft survival to conventional KDPI < 0.20 organs.

**Conclusion:**

EDAM provides an equitable, donor-centric framework for assessing kidney quality. Although these categorical thresholds were derived in a data-driven manner within the US system and require external validation in international cohorts, EDAM’s ability to safely expand the low-risk pool without compromising outcomes suggests it could significantly refine allocation policy and enhance fairness in kidney transplantation.

## Graphical abstract

**Figure:**
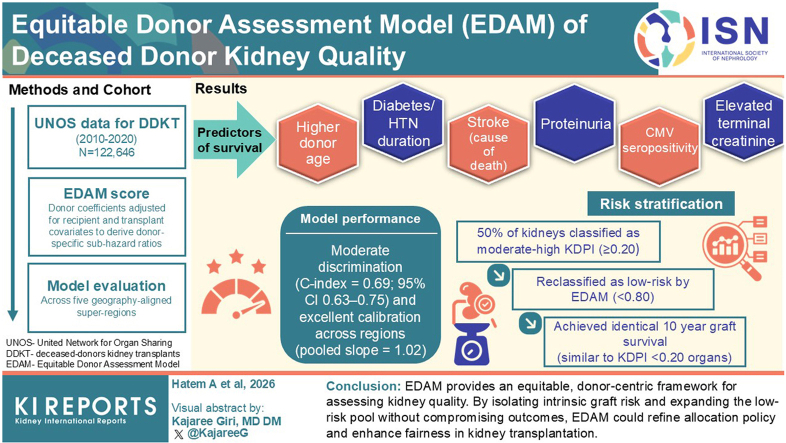

Kidney transplantation remains the definitive treatment for end-stage kidney disease; however, the mismatch between organ demand and supply continues to widen.^1^ Ensuring fair access to scarce donor kidneys while maximizing graft survival is critical both for individual patient outcomes and for maintaining public trust in the allocation system.

The KDPI has standardized donor assessment by predicting “overall graft survival”; however, this composite end point blends intrinsic graft quality with recipient survival risk.^2^ As a result, high-quality kidneys transplanted into recipients with higher comorbid mortality may be misclassified as marginal and diverted from optimal use thus contributing to unnecessary discard and exacerbating disparities, particularly among minority populations whose nonorgan factors can adversely affect posttransplant survival.^3^ Recent Scientific Registry of Transplant Recipients analyses have highlighted calibration weaknesses in the original KDPI model at the extremes of donor risk, underscoring the need for improved donor-specific evaluation tools.^4^

To address these limitations, we developed the EDAM, a donor-focused risk stratification tool that isolates intrinsic graft failure risk from recipient factors. EDAM employs Fine-Gray competing-risks regression to model pure graft failure, deliberately excludes donor ethnicity, and incorporates key donor demographics and clinical variables. By decoupling donor-specific risk from recipient mortality, EDAM is designed to refine organ quality assessment and complement recipient-centric tools such as the estimated posttransplant survival score within allocation algorithms.

In this study, we used UNOS data from 2010 to 2020 to develop and internally validate EDAM. Our goal was not to generate individual patient-outcome predictions, but to establish a robust framework for ranking donor kidneys into clinically meaningful risk categories based solely on intrinsic characteristics. We hypothesized that EDAM will more accurately reflect graft failure risk than composite models and thereby improve equity and efficiency in kidney allocation.

## Methods

To provide a more precise evaluation of donor risk, EDAM was developed using Fine-Gray competing-risks regression. In this framework, the primary outcome was graft failure, defined as the loss of kidney function requiring dialysis or retransplantation, while death with a functioning graft was modelled as a competing event. The model incorporated key donor characteristics with demonstrated biological and clinical plausibility, and coefficients were adjusted for recipient and transplant covariates to isolate donor-specific effects. SHRs and 95% confidence intervals (CIs) were estimated to quantify the relative contribution of each donor variable to death-censored graft failure.

### Study Design and Data Source

This retrospective cohort study used data from the UNOS repository, focusing on kidney transplant operations recorded between January 1, 2010, and January 1, 2020. Follow-up data were collected through January 1, 2025, for a minimum of 5 years, allowing a comprehensive assessment of graft survival over time. Donor characteristics known to affect graft survival were analyzed, including donor age, sex, body mass index, cause of death, serum creatinine, history of diabetes, history of hypertension CMV status, presence of proteinuria, non–heart-beating donor status, *en bloc* kidney and donor blood group.^1^, ^2^, ^3^, ^4^, ^5^ UNOS collects “protein in urine” simply by recording the result (yes/no/unknown) of the terminal urinalysis performed on a deceased donor immediately before cross-clamp, storing it as a 1-character categorical field in the Deceased Donor Registration.

When establishing the EDAM, we adjusted for recipient- and transplant-related factors, but chose to incorporate in the index itself only factors that characterized the donor kidney. The transplant and recipient factors that were used for adjustment included the following: HLA mismatch, cold ischemia time, calculated panel reactive antibody, recipient age, sex, ethnicity, diabetes, time on the waiting list, recipient CMV status, and dialysis before transplant. For each donor, the terminal serum creatinine value recorded in DonorNet at retrieval was used. Earlier intensive care unit creatinine values were not consistently available across centers. The model was fitted to estimate the relative rate of graft failure independently associated with each donor factor, with these adjustments ensuring the accurate calculation of donor factor coefficients.

### Participant Selection and Missing Data

Recipients of kidney transplants from deceased donors were included, whereas multiorgan transplant recipients were excluded. Cases with missing data were also excluded.

### Statistical Analysis

#### Competing-Risks Regression

A competing-risks regression model was constructed following the Fine-Gray subdistribution hazard approach. A regression model for competing risks was developed using jack-knife pseudo-observations of the Aalen-Johansen cumulative incidence function at 5 years posttransplant. These pseudo-observations were used to generate a risk score. These pseudo-observations were regressed on the candidate predictors using a generalized linear model with a complementary log-log link function and robust standard errors, approximating the Fine-Gray subdistribution hazard. The exponentiated coefficients from this model were interpreted as SHRs.^5^ The choice of 5 years for generating pseudo-observations was driven by the comprehensive nature of our dataset, which used UNOS data from 2010 to 2020 with follow-up extending through January 1, 2025, thereby ensuring a minimum of 5 years of follow-up for all included transplants. This duration allowed for a robust and data-supported assessment of graft survival over a clinically meaningful timeframe. We assessed potential nonlinearity using visual checks. For continuous predictors, we first inspected visual diagnostics (LOESS or partial residual plots of outcome vs. predictor and calibration-by-deciles). If curvature is suggested, we performed a restricted cubic spline sensitivity. Recipient and transplant factors (including recipient age, sex, ethnicity, diabetes, dialysis status, HLA mismatch, cold ischemia time, and waiting time) were entered in the model as covariates to adjust donor coefficients, ensuring that the estimated associations reflected intrinsic donor kidney quality independent of recipient- or transplant-related influences. The final EDAM index retained only donor variables, with their coefficients derived from this adjusted model. Predictive performance and discrimination were then evaluated in recipient-adjusted analyses to confirm its robustness across varying recipient and transplant profiles. Initially, univariate competing-risks regressions were performed for each variable of interest; variables with a P-value < 0.05 were included in the multivariable model.

#### Model Optimization and Predictor Selection

To mitigate overfitting and collinearity, we used LASSO penalization with the penalty chosen by the Bayesian Information Criterion. Multilevel factors were penalized at the group level. The final analysis model was then refit on the LASSO-selected set (postselection refit) using the same link or variance specification to produce the reported coefficients and intervals.

#### Internal-External Cross-Validation

Model transportability and optimism were evaluated using internal-external validation across geography-aligned super-regions with calibration slope and intercept as the primary metrics; fold-specific slopes were pooled using random-effects meta-analysis.^6^^,^^7^ We aggregated the 11 UNOS regions into 5 geography-aligned super-regions to create realistic domain shifts while preserving adequate sample size in each held-out fold. This grouping was prespecified and reflects coherent care ecosystems and referral or offer pathways rather than statistical convenience. Specifically, we combined New England and Mid-Atlantic states (UNOS 1, 9, and 10) into a Northeastern super-region; clustered the Upper Midwest (UNOS 2 and 6); retained a broader Midwest and Plains block (UNOS 3); grouped Western programs with similar logistics and donor management (UNOS 4 and 8); and combined the South and Southeast into a single super-region (UNOS 5, 7, and 11). This structure mirrors how organs and clinical practices vary across the US, yielding 5 folds that are both geographically sensible and statistically stable for calibration estimation. In each iteration, we trained on 4 super-regions and validated on the fifth, estimating calibration intercept (ideal 0) and slope (ideal 1) by regressing observed pseudo-values on the model’s linear predictor. Fold-specific slopes were then pooled with random-effects meta-analysis to summarize transportability.

#### Risk Stratification

The coefficients of the donor factors were used to calculate the EDAM score, without normalization or standardization. Donors were subsequently stratified into categories according to this score, allowing a detailed differentiation of donor risk profiles.

#### Model Performance

Harrell's C-index was used to assess the model's discrimination power across the entire follow-up period. It was calculated using inverse probability of censoring weighting, with Kaplan-Meier estimates for the censoring distribution. This discrimination measure ranges from 0.5 (no better than chance) to 1.0 (perfect discrimination).^8^^,^^9^ We evaluated calibration of the graft-failure risk by grouping recipients into 10 equally sized categories according to their predicted risk and, within each group, comparing the average predicted probability with the average observed incidence (estimated using pseudo-observations). We then plotted these 10 points against the 45° line of perfect agreement to produce a decile-based calibration curve.

To summarize calibration numerically, we fit a linear calibration model of observed versus predicted risk across deciles, reporting the intercept (indicating overall bias, or “miscalibration-in-the-large”) and the slope (indicating whether estimations are too extreme or too regressed toward the mean). In a perfectly calibrated model, the intercept equals 0 and the slope equals 1.

#### Validation Using Bootstrap Method

Bootstrapping was employed to robustly estimate the C-index's uncertainty, thereby providing a reliable measure of uncertainty for the model's discriminative ability. A total of 50 bootstrap replications were performed by sampling with replacement from the original dataset, ensuring that each bootstrap sample maintained the same size as the full cohort. For each replication, the Harrell's C-index was calculated, accounting for time-to-event data, censoring status, and inverse probability of censoring weights. It was then precisely extracted for each bootstrap replication by calculating the midpoint of its reported symmetric CI bounds. The final C-index uncertainty was then derived using the bias-corrected bootstrap method.

#### Subgroup Analyses

To explore demographic influences on graft outcomes, we conducted subgroup analyses stratified by race (White vs. non-White) and age (≥ 65 vs. < 65 years), examining relative SHRs within these subpopulations.

#### Risk Stratification and Cumulative Incidence Function Analysis

To assess the relationship between our EDAM risk index and 5-year graft failure, we modelled the cumulative incidence using a generalized linear framework with a complementary log-log link. This approach is well-suited to time-to-event data with competing risks, and it allowed us to express the risk in terms of SHRs. We used robust standard errors to account for potential heteroskedasticity.

We then estimated the predicted 5-year cumulative incidence across a range of EDAM scores—from 0.5 to 1 in small increments—to capture how risk increases continuously with higher EDAM values. These model-based estimations were plotted against the EDAM score, providing a clear visual illustration that the risk of graft failure rises steadily as the donor risk index increases.

In addition to modelling the 5-year cumulative incidence, we repeated the analysis for a 10-year time horizon. Using the same generalized linear model with a complementary log-log link and robust standard errors, we estimated predicted cumulative incidence values across the full range of EDAM scores. The resulting 10-year risk estimates increased steadily with higher EDAM values, mirroring the pattern observed at 5 years and reinforcing the index's ability to discriminate donor risk over both intermediate and long-term follow-up

The continuous donor-risk measure from the competing-risks subdistribution hazard of the donor factors—formalized as the EDAM score—was used to divide donors into 5 categories reflecting ascending gradients of donor risk.

To account for competing mortality (death with a functioning graft), the cumulative incidence function for graft failure was calculated within each category. We subsequently compared the cumulative incidence functions across the categories using Gray’s test, which provides a nonparametric assessment of differences in cumulative incidence in the presence of competing events. Because Stata does not have a built-in Gray’s test, an external software (the cuminc package in R [R Foundation for Statistical Computing, Vienna, Austria]) was employed for this purpose. Statistical significance was defined at *P* < 0.05.

#### Comparative Survival Analysis of KDPI Versus EDAM Low-Risk Cohorts

We divided donor kidneys into 2 mutually exclusive low-risk groups. The standard low-risk group included all kidneys with KDPI < 0.20 (the lowest 20th percentile under Kidney Allocation System). The extended low-risk group comprised those additional kidneys that EDAM identified as low risk (EDAM < 0.80) but did not meet the KDPI criterion (i.e. KDPI ≥ 0.20). Recipients were followed up from transplantation until graft failure or administrative censoring at 10 years. Cumulative incidence curves were generated for each cohort, and differences in graft-failure distributions were assessed visually and using the log-rank test.

### Statistical Software

All statistical analyses were performed using Stata 17 (StataCorp, College Station, TX), which provided the necessary tools for advanced survival analysis and handling of competing risks scenarios.^10^

### Ethical Approval

This study used deidentified data from UNOS. Research using registry, deidentified data, such as that from UNOS, does not constitute "human subjects research" as defined by applicable regulations. Therefore, formal institutional review board review and approval were not required for this study. The data were analyzed in compliance with all applicable data use agreements and ethical guidelines for research using registry data.

## Results

### Participant Selection

The initial cohort included 126,288 recipients of kidney-only transplants from deceased donors during the study period. A total of 3642 recipients (2.88%) were excluded because of incomplete data in key variables, resulting in a final analytic cohort of 122,646 transplants. Specifically, 2109 recipients were excluded because of missing data on waiting time to transplantation, and 1533 were excluded because of missing information on HLA mismatch or cold ischemia time. Given the low proportion of missingness (< 3%) and its confinement to essential transplant covariates, a complete-case analysis was performed to preserve internal consistency and prevent the introduction of model instability through imputation. Sensitivity checks using multiple imputations in a subset of cases yielded similar coefficient estimates, supporting the robustness of the complete-case approach.

### Baseline Demographic and Clinical Characteristics

Baseline characteristics of 122,646 donor-recipient pairs are summarized in Table 1. Donors had a mean age of 37.8 years (SD: 15.8); 72.5% had no history of hypertension, 26.8% had hypertension of varying duration, and 7.7% had diabetes. Proteinuria was present in 46.2% of donors, and 60.7% were CMV seropositive. The leading causes of donor death were anoxia (39.5%), trauma (33.3%), and stroke (26.7%). Recipients had a mean age of 51.5 years (SD: 15.2); 40.1% were female, 34.9% had diabetes, and most were White (39.8%), Black (32.9%), or Hispanic (18.3%). The mean waiting time before transplantation was 1480 days (SD: 1127). Key transplant characteristics, including the distribution of HLA mismatches, calculated panel reactive antibody (mean: 23.9% ± 36.4%), and mean cold ischemia time (17.4 ± 8.8 hours) are presented in detail in Table 1.

**Table 1:** Baseline characteristics of the analysis cohort

Variable	Category / Summary	*n* (%) or mean ± SD (range)
Donor factors		
Non–heart-beating donor	No	99,316 (81.0)
	Yes	23,330 (19.0)
Proteinuria in urine	No	65,105 (53.1)
	Yes	56,720 (46.2)
	Unknown	821 (0.7)
Donor CMV serology	Negative	48,158 (39.3)
	Positive	74,484 (60.7)
	Unknown	4 (0.0)
History of hypertension	No	88,881 (72.5)
	Yes	32,906 (26.8)
	Unknown	859 (0.7)
Hypertension duration	No	88,881 (72.5)
	0–5 yrs	16,245 (13.2)
	6–10 yrs	6129 (5.0)
	> 10 yrs	6357 (5.2)
	Duration unknown	5034 (4.1)
Diabetes duration	No	113,234 (92.3)
	0–5 yrs	4212 (3.4)
	6–10 yrs	1640 (1.3)
	> 10 yrs	1874 (1.5)
	Duration unknown	1686 (1.4)
Cause of death	Anoxia	48,487 (39.5)
	Stroke	32,739 (26.7)
	Trauma	40,893 (33.3)
	Tumor	527 (0.4)
Donor creatinine (mg/dl)		1.20 ± 1.07 (0.02–37.0)
Donor age (yrs)		37.75 ± 15.82 (0–88)
Donor BMI (kg/m^2^)		27.74 ± 7.01 (7.75–74.36)
*En bloc* kidneys (Yes vs. no)	Yes	2036 (1.66)
	No	120,620 (98.34)
Transplant factors		
HLA mismatch (level 0–6)	0	7247 (5.9)
	1	1628 (1.3)
	2	5694 (4.6)
	3	16,761 (13.7)
	4	33,448 (27.3)
	5	39,193 (32.0)
	6	18,675 (15.2)
Calculated PRA (%)		23.85 ± 36.39 (0–99.9996)
Cold ischemia time (h)		17.39 ± 8.80 (0.01–99)
Recipient factors		
Recipient age (yrs)		51.53 ± 15.24 (0–89)
Recipient gender	Female	49,155 (40.1)
	Male	73,491 (59.9)
Recipient diabetes	No	79,657 (65.1)
	Yes	42,752 (34.9)
	Missing/unknown	237 (0.2)
Recipient ethnicity	White	48,814 (39.8)
	Black	40,299 (32.9)
	Hispanic	22,482 (18.3)
	Asian	8530 (7.0)
	American Indian	1248 (1.0)
	Pacific Islander	569 (0.5)
	Multiracial	704 (0.6)
Dialysis at transplant	No	13,096 (10.7)
	Yes	109,550 (89.3)
Previous transplants	0	106,943 (87.2)
	1	13,678 (11.1)
	2	1699 (1.4)
	3	249 (0.2)
	≥ 4	77 (0.1)
Days on waitlist		1480.08 ± 1126.69 (0–15,395)
Recipient CMV serology	Negative	39,011 (31.8)
	Positive	82,754 (67.5)
	Unknown	879 (0.7)

BMI, body mass index; CMV, cytomegalovirus; HLA, human leukocyte antigen; PRA, panel reactive antibody.

### Predictors of Graft Survival Among Deceased Donor Kidney Transplants

A refined multivariable competing-risks regression model identified several donor factors significantly associated with graft survival (Table 2). Each additional year of donor age was associated with a 1.3% increase in the risk of graft failure (SHR = 1.0127, 95% CI: 1.011–1.014, *P* < 0.001). With anoxia as the reference category, donors who died of stroke had a significantly higher risk (SHR = 1.125, 95% CI: 1.073–1.181, *P* < 0.001), whereas those who died of trauma (SHR = 0.970, 95% CI: 0.926–1.017, *P* = 0.203) or tumor (SHR = 1.240, 95% CI: 0.958–1.606, *P* = 0.103) did not differ significantly from the reference. Donor body mass index was inversely associated with graft failure (SHR = 0.993, 95% CI: 0.990–0.996, *P* < 0.001), whereas positive CMV serology conferred a modest but significant increase in risk (SHR = 1.072, 95% CI: 1.031–1.114, *P* < 0.001).

**Table 2:** Results of generalized linear model for risk of graft failure using donor characteristics (adjusted for recipient and transplant factors)

Variable	Subcategory / Comparison	SHR (95% CI)	*P*-value
Donor characteristics			
Sex (male vs. female)		0.934 (0.898–0.971)	0.001
Age (/yr increase)		1.013 (1.011–1.014)	< 0.001
Cause of death (Ref: anoxia)	Stroke	1.125 (1.073–1.181)	< 0.001
	Trauma	0.970 (0.926–1.017)	0.203
	Tumor	1.240 (0.958–1.606)	0.103
BMI (per kg/m^2^)		0.993 (0.990–0.996)	< 0.001
CMV serology (positive vs. negative)		1.072 (1.031–1.114)	< 0.001
History of hypertension (Ref: no)	0–5 yrs	1.178 (1.114–1.246)	< 0.001
	6–10 yrs	1.187 (1.093–1.289)	< 0.001
	> 10 yrs	1.343 (1.242–1.452)	< 0.001
	Duration unknown	1.191 (1.086–1.307)	< 0.001
History of diabetes (Ref: no)	0–5 yrs	1.247 (1.138–1.367)	< 0.001
	6–10 yrs	1.607 (1.414–1.827)	< 0.001
	> 10 yrs	1.915 (1.709–2.145)	< 0.001
	Duration unknown	1.180 (1.017–1.370)	0.029
Serum creatinine (per mg/dl)		1.022 (1.005–1.040)	0.013
Proteinuria (Ref: no)	Unknown	0.944 (0.745–1.195)	0.632
	Yes	1.045 (1.006–1.085)	0.022

BMI, body mass index; CI, confidence interval; CMV, cytomegalovirus; Harrell’s C: Harrell’s Concordance Index; SHR, subhazard ratio.

Donors with a history of hypertension exhibited progressively higher risks with longer duration (SHR = 1.178 for 0–5 years, 1.187 for 6–10 years, 1.343 for > 10 years, and 1.191 for unknown duration; all *P* < 0.001). Similarly, donor diabetes was associated with increasing risk by duration (SHR = 1.247 for 0–5 years, 1.607 for 6–10 years, and 1.915 for > 10 years; all *P* < 0.001), whereas an unknown duration yielded SHR = 1.180 (*P* = 0.029). Higher donor serum creatinine was independently associated with poorer graft outcomes (SHR = 1.022, 95% CI: 1.005–1.040, *P* = 0.013), and the presence of donor proteinuria was linked to a small but significant risk increase (SHR = 1.045, 95% CI: 1.006–1.085, *P* = 0.022). Notably, male donor sex was associated with a lower subhazard compared with female donors (SHR = 0.934, 95% CI: 0.898–0.971, *P* = 0.001). Full model estimates prior to lasso selection are presented in Supplementary Table S1. Comparison of the donor-only and adjusted models (Supplementary Table S2) demonstrated that adjustment for recipient and transplant factors minimally altered the donor-specific coefficients, confirming that EDAM’s predictive structure is predominantly donor-driven and robust to downstream recipient-level variation.

### Assessment of Interaction Between Cold Ischemia Time and Non–Heart Beating Donors

Including donor type and its interaction with cold ischemia time did not materially change model performance. In the fully adjusted model, non–heart-beating (donation after circulatory death [DCD]) versus donation after brain death was not associated with higher risk (SHR = 0.98; 95% CI: 0.86–1.10; *P* = 0.715), and the DCD × cold ischemia time interaction was not significant (/h interaction SHR = 1.003; 95% CI: 0.997–1.009; *P* = 0.286). Cold ischemia time, nonetheless, retained a small independent effect (/h SHR = 1.006; *P* < 0.001).

### Model Performance

Harrell’s C-index was used to evaluate the discriminative ability of our final model, both in the overall cohort and within key subgroups. Overall, the model achieved a C-index of 0.69, indicating a moderate level of discrimination.

When stratified by recipient ethnicity, the C-index was 0.69 among White recipients and 0.67 among the non-White recipients, suggesting similar or slightly better model performance in these subgroups. Age-based stratification (< 65 vs. ≥ 65 years) showed C-index values of 0.69 for younger recipients and 0.68 for older recipients, indicating consistent discrimination across these age categories.

Overall, these findings suggest that the model retains a moderate predictive ability across diverse recipient populations and age groups.

**Figure 1:**
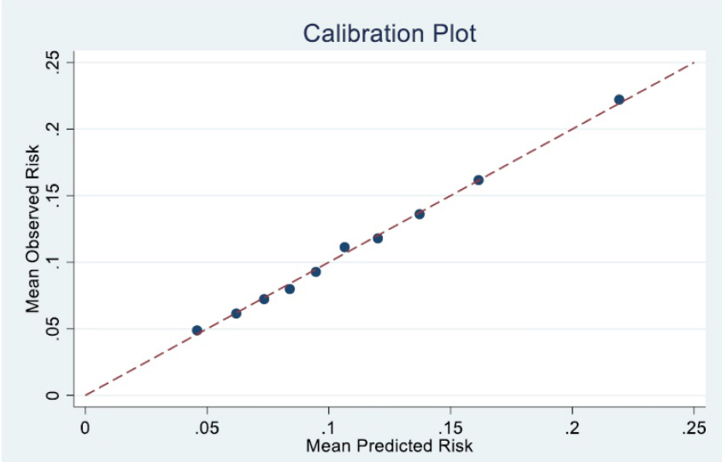
Calibration plot for the generalized linear model.

In Figure 1, we show the calibration plot for the generalized linear model. The calibration slope is 1.009 (95% CI: 0.96–1.05). The intercept of the calibration curve is −0.001.

### Risk Stratification Using EDAM and Cumulative Incidence Function Analysis

The final equation of the EDAM risk score based on the generalized linear model is as follows: “EDAM = 1 − exp{− exp[0.0126 × (Donor Age) + 0.118 × (Cause of Death = Stroke) − 0.031 × (Cause of Death = Trauma) + 0.215 × (Cause of Death = Tumor) − 0.0066 × (Donor body mass index) + 0.070 × (CMV Serology = Positive) + 0.164 × (Hypertension 0–5 years) + 0.172 × (Hypertension 6–10 years) + 0.295 × (Hypertension >10 years) + 0.175 × (Hypertension Duration Unknown) + 0.221 × (Diabetes 0–5 years) + 0.475 × (Diabetes 6–10 years) + 0.650 × (Diabetes >10 years) + 0.165 × (Diabetes Duration Unknown) + 0.022 × (Serum Creatinine) + 0.044 × (Proteinuria = Yes) − 0.069 × (Donor Sex = Male)]}”.

**Figure 2:**
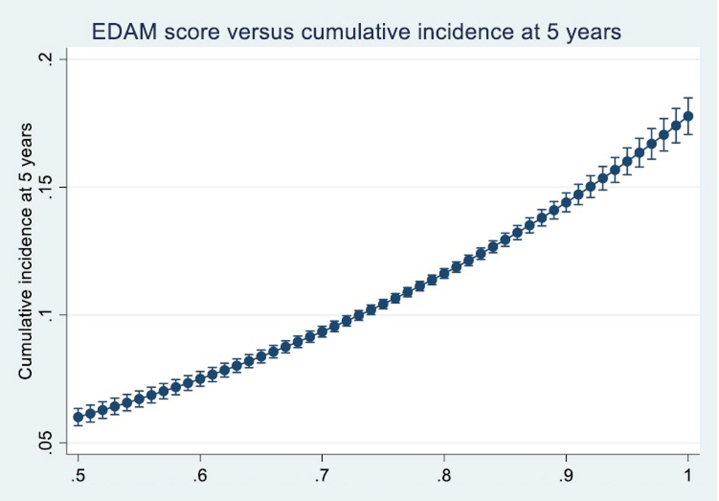
EDAM score plotted against cumulative incidence function at 5 years.

The EDAM score ranged from 0.5122398 to 0.9983422. In Figure 2, we display the predicted 5-year cumulative incidence of graft failure as a function of the EDAM score. As the EDAM score increases, the graph shows a steady, monotonic rise in the cumulative incidence, demonstrating that higher donor risk, as quantified by EDAM, is associated with a greater risk of graft failure. This clear gradient supports the potential application of the EDAM score in the kidney allocation process.

**Figure 3:**
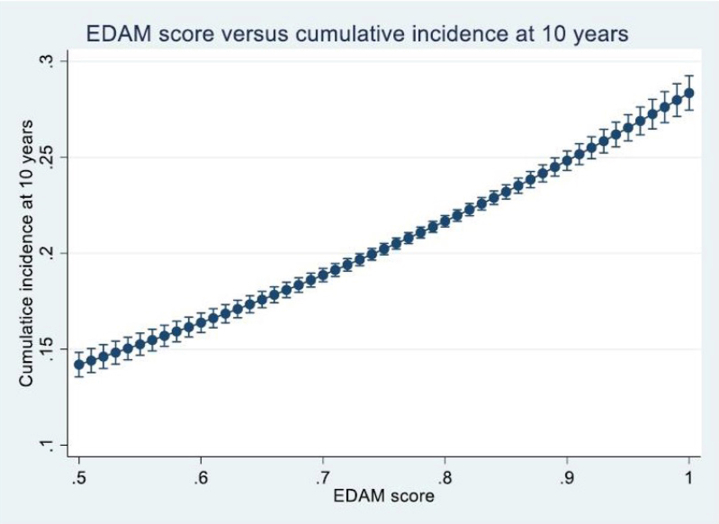
EDAM score plotted against the cumulative incidence function at 10 years.

**Figure 4:**
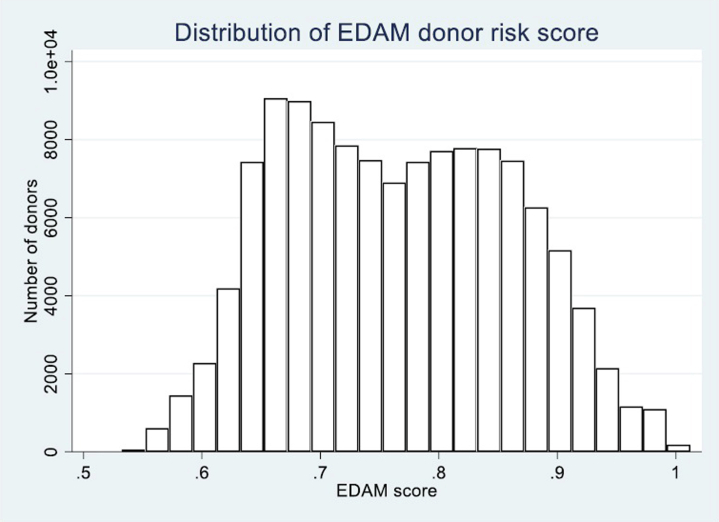
Distribution of the EDAM score.

In Figure 3, we present the analogous relationship for a 10-year time horizon. Here too, as the EDAM score increases, the predicted long-term risk of graft failure increases, further validating the prognostic performance of the EDAM index over both intermediate and long-term follow-up. These findings suggest that the continuous EDAM score could be integrated into allocation schemes, as a continuous variable to enhance donor-recipient matching and improve overall transplant outcomes. In Figure 4, we show the distribution of the EDAM score.

The distribution of the EDAM score was highly skewed, with majority of donors concentrated in the 0.7 to 0.9 range, as demonstrated in Figure 4. A centile analysis revealed that donors with an EDAM < 0.8 comprised a large and relatively homogeneous group; whereas the risk of graft failure, as estimated by the predicted cumulative incidence, increased markedly above an EDAM of 0.8. The centile analysis was performed by partitioning the full range of EDAM scores into 0.05-unit intervals and calculating the observed 10-year graft-failure incidence, followed by visual inspection of these interval-specific risk estimates.

**Figure 5:**
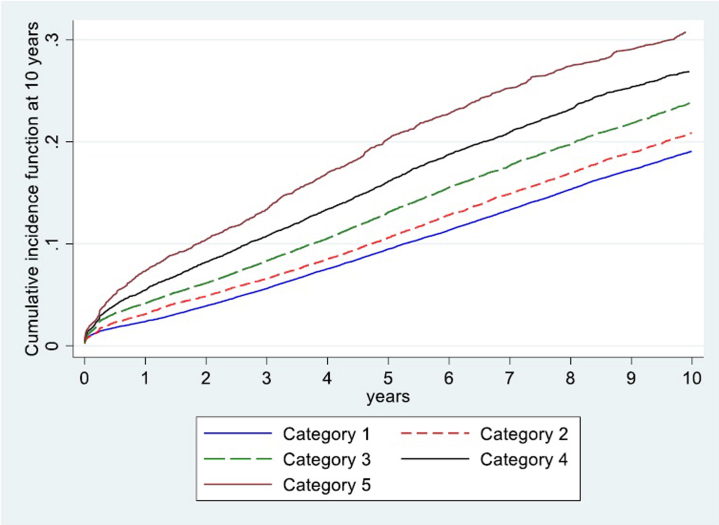
Comparison of the cumulative incidence function at 10 years across the 5 categories.

Based on these observations, we defined 5 EDAM categories. Donors with an EDAM score < 0.8 were classified as lower risk (category 1), whereas the higher-risk segment was subdivided into 4 groups: those with scores between 0.8 and 0.85 (category 2), between 0.85 and 0.9 (category 3), those with scores between 0.9 and 0.95 (category 4), and those with scores ≥ 0.95 (category 5). This data-driven stratification not only reflects the empirical distribution of EDAM but also captures the clear, stepwise increase in risk observed both in the margins plot (Figure 3) and in the cumulative incidence functions (Figure 5), thereby providing reproducible and clinically interpretable risk groupings, where the terms “low” and “high” risk denote relative hazard gradations derived from the observed data.

In Figure 5, we illustrate the cumulative incidence functions of graft failure across the 5 categories. The increasing trend in graft failure incidence with higher categories aligns with the generalized linear model findings, providing a cohesive narrative on the impact of donor health disparity on transplantation success. Gray`s test showed a *P* value < 0.05, indicating significant statistical differences between the groups.

### Bootstrap Validation

The discriminative ability of the model, as assessed by Harrell's C-index in the full cohort, was 0.69. Bootstrap analysis, employing 50 replications with sampling with replacement, yielded a bootstrap standard error of 0.04 and an estimated bias of −0.01. The 95% bias-corrected bootstrap CI for the C-index was 0.66 to 0.77. The results of the bootstrap analysis among the key subgroups are shown in Table 3.

**Table 3:** Discriminative Performance of the Final Model Evaluated by Harrell’s C-index, and validated using bootstrap analysis

Population/subgroup	C-index (95% CI)
Overall cohort	0.69 (0.63–0.75)
Recipient ethnicity	
White	0.69 (0.63–0.74)
Non-White	0.67 (0.59–0.74)
Recipient age	
< 65 yrs	0.70 (0.62–0.74)
≥ 65 yrs	0.69 (0.60–0.79)

CI**,** confidence interval.

### Results of the Comparative Survival Analysis of KDPI Versus EDAM Low-Risk Cohorts

**Figure 6:**
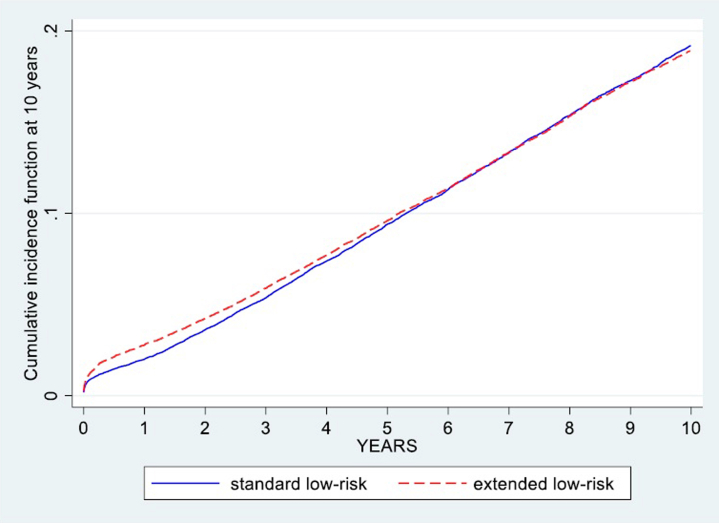
Ten-year cumulative incidence of graft failure in the standard low-risk group (KDPI < 0.20) versus the extended low-risk group (kidneys classified low-risk by EDAM only and not by KDPI: EDAM < 0.80 and KDPI ≥ 0.20).

**Figure 7:**
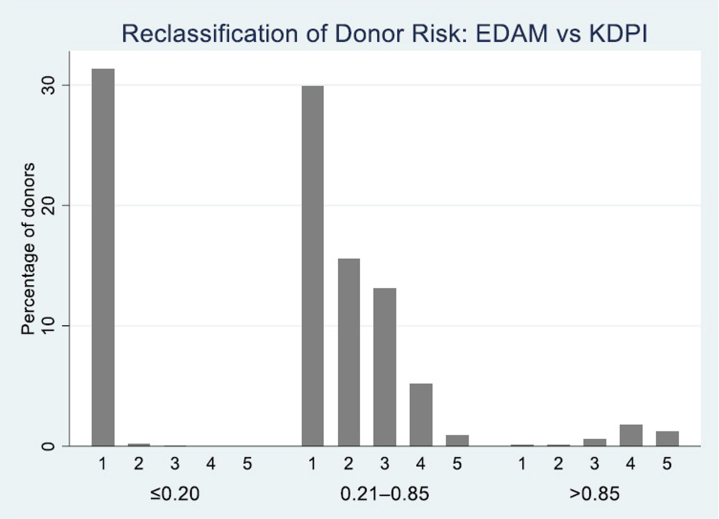
Mapping of KDPI versus EDAM.

In our cohort of 75,172 donor kidneys classified as low risk by EDAM (EDAM < 0.80), 38,389 (51.1%) met the conventional KDPI < 0.20 criterion, whereas the remaining 36,783 (48.9%) formed the “extended” low-risk group (EDAM < 0.80 & KDPI ≥ 0.20). As shown in Figure 6, the 10-year cumulative incidence curves for graft failure in these 2 groups were virtually superimposable, indicating no detectable difference in long-term graft survival between kidneys classified as low risk by KDPI and those reclassified as low risk by EDAM. This demonstrates that EDAM effectively broadens the definition of low-risk donors without compromising graft outcomes, supporting its potential utility for expanding organ utilization. To visualize how the EDAM model reclassifies donor risk compared with the KDPI, donors were grouped by KDPI categories (≤ 0.20, 0.21–0.85, and > 0.85) and plotted against EDAM quintiles

(Figure 7). Although EDAM and KDPI were broadly correlated, EDAM provided markedly finer granularity in risk assessment. Within the large mid-range KDPI group (0.21–0.85), which included most donors, EDAM redistributed kidneys across all 5 quintiles, identifying both lower- and higher-risk subsets that were indistinguishable under KDPI alone.

Among kidneys with KDPI > 0.85, approximately one-third were classified by EDAM into intermediate-risk quintiles (Q3–Q4), suggesting potential for selective use of higher-KDPI organs, whereas a small proportion of kidneys with KDPI ≤ 0.20 were assigned to higher-risk EDAM categories, reflecting residual donor-level risk not captured by KDPI.

Overall, the mapping demonstrates that EDAM refines donor quality assessment beyond the broad KDPI thresholds, providing a more continuous and clinically informative gradient of graft-failure risk.

### Results of the Internal External Validation

Internal-external cross-validation across the 5 predefined super-regional folds confirmed the stability and generalizability of the EDAM model. Calibration intercepts were uniformly close to 0 (range −0.015 to 0.009), indicating minimal systematic bias. Calibration slopes ranged from 0.866 to 1.186 (SE: 0.024–0.060), demonstrating consistent agreement between predicted and observed risks across geographically distinct validation sets.

When pooled using a random-effects meta-analysis, the summary calibration slope was 1.02 (95% CI: 0.94–1.10), and the pooled intercept was −0.002 (95% CI: −0.012 to 0.008), both

consistent with near-perfect calibration. These results support the model’s robustness and transportability, indicating that no regional recalibration was required and justifying the final model’s estimation using the complete national dataset.

## Discussion

Our study introduces EDAM, which employs a competing-risks regression framework based exclusively on donor characteristics. Unlike traditional models such as KDPI, which rely on overall graft survival endpoints, or the European KTOP model, which includes both donor and recipient variables and is limited to donation after brain death donors, EDAM quantifies the donor’s specific contribution to graft failure.^1^^,^^11^ Although the model adjusts for transplant and recipient factors to ensure accurate estimation of donor-specific hazard ratios, it primarily focuses on donor-derived risk by treating death with a functioning graft as a competing event. This approach isolates the intrinsic risk associated with the donor kidney, providing a more refined framework for risk stratification in kidney transplantation. To our knowledge, this is the first study to apply such a methodologically advanced competing-risks approach for the stratification of deceased donors, marking a significant advancement in the field of transplant medicine.

It is important to emphasize that though the categories offer a simplified framework for risk communication and initial stratification, the EDAM score itself is a continuous measure of risk. The categories serve as clinically relevant tiers designed to group donors with similar risk profiles, rather than representing infinitely precise, rigid boundaries where risk instantaneously changes. The “marked” increase in risk around specific thresholds reflects the overall trend observed in the data, where a step-wise progression of risk becomes apparent at these score intervals. The robustness of these thresholds is supported by the statistically significant separation of cumulative incidence functions (Gray's test, *P* < 0.05), indicating that the distinctions between these risk groups are indeed real within our large cohort. Although minor measurement variations can lead to recategorization, the primary utility of EDAM lies in its ability to stratify donors into broad risk groups that facilitate discussions and decisions, recognizing that clinical judgment always accompanies any score-based allocation.

Although our analysis timeframe (2010–2020) reflects more recent practices than those used in creating KDPI (1995–2005), we acknowledge that certain current procedures (e.g., machine perfusion, normothermic organ harvesting) remain unaccounted for. Variables such as glomerulosclerosis and warm ischemia time were excluded because of > 50% and > 80% missing data, respectively. Moreover, real-time biopsy findings (e.g., percentage of glomerulosclerosis) often become available only late in the allocation process, risking potential delays. Future efforts to improve data completeness and streamline the availability of histopathological findings could further refine donor stratification models.^12^, ^13^, ^14^, ^15^, ^16^

Because normothermic regional perfusion is increasingly used in DCD procurement, we assessed whether donor type and a DCD–cold-ischemia interaction were required in EDAM. In our national cohort, neither DCD status nor its interaction with cold ischemia time was independently associated with death-censored graft failure after adjustment for core donor factors (age, comorbidities, creatinine, proteinuria) and transplant factors (HLA mismatch, calculated panel reactive antibody, dialysis, wait-time). Two considerations likely explain this as follows: (i) normothermic regional perfusion mitigates warm-ischemia injury and may narrow outcome differences between DCD and donation after brain death; and (ii) our model already incorporates cold ischemia time—the principal modifiable ischemic exposure—so residual DCD effects may be minimal once cold ischemia time and donor biology are accounted for. As standardized normothermic regional perfusion coding becomes more widely available, EDAM can be updated or recalibrated to include a direct normothermic regional perfusion indicator; however, current internal-external validation showed stable calibration across regions, supporting use of the present model without DCD-specific adjustment.^16^

Our findings reveal that KDPI’s composite end point—combining graft failure with patient mortality—can misclassify high-quality kidneys and deepen disparities for minority recipients, who face higher posttransplant mortality because of socioeconomic and access barriers.^3^^,^^4^ By using a competing-risks framework to isolate donor-derived failure risk, EDAM offers a more precise assessment of intrinsic kidney quality, unconfounded by recipient survival factors.^17^, ^18^, ^19^, ^20^ Moreover, EDAM’s discrimination (C-index: 0.69) exceeds the reported performance of KDPI for overall graft survival (C-index: 0.60), demonstrating at least equivalent predictive ability while providing a fairer basis for allocation.^21^, ^22^, ^23^, ^24^, ^25^, ^26^

In line with a broader commitment to fairness, EDAM does not include donor ethnicity as a predictive variable. The donor ethnicity variable was removed from the KDPI in October 2024.^26^ This decision was guided by a desire to minimize biases and align with policy changes by the UNOS, which emphasize equity in organ allocation. Excluding donor ethnicity in EDAM ensures that kidneys are evaluated solely based on objective measures of organ quality, aligning seamlessly with UNOS guidelines designed to reduce systemic biases in transplantation.^26^

The relationship between donor age and graft failure can be nonlinear; very young donors often require *en bloc* transplantation because of small kidney size and delicate vasculature. In our cohort, *en bloc* cases were rare (1.64%) and did not reach statistical significance (*P* > 0.05), likely reflecting limited power rather than lack of clinical relevance. Therefore, although nonlinear size effects may exist, we retained linear terms to preserve EDAM’s balance of predictive accuracy and interpretability for broad donor assessment.^27^ Because procurement biopsies are not uniformly performed across transplant systems and their predictive value remains uncertain, EDAM provides a reproducible alternative for quantifying donor kidney quality using routinely available clinical variables. Therefore, rather than replacing histological assessment where it is indicated, EDAM offers a complementary data-driven framework that may support more consistent decision-making and reduce variability in organ acceptance practices.

Our findings show that by adopting EDAM to define low-risk kidneys (EDAM < 0.80), the pool of organs eligible for longevity matching more than doubles compared with the current KDPI-based cutoff; yet these additional kidneys demonstrate virtually identical 10-year graft-failure rates (Figure 6). In practical terms, this means transplant programs could offer a far greater number of high-quality organs preferentially to the healthiest candidates without compromising outcomes. A larger longevity-matched cohort is likely to shorten wait times for younger, lower-risk recipients—reducing time on dialysis, pretransplant morbidity, and potentially improving posttransplant conditioning—while preserving overall graft survival. The choice of EDAM < 0.80 as the low-risk cutoff was data-driven, representing the point where graft survival matched that of the KDPI < 20% benchmark. Although a more conservative threshold (e.g., 0.75) would yield slightly higher survival estimates, it would reduce organ availability. The optimal policy threshold may therefore be tuned to local priorities, balancing maximization of organ use with acceptable post-transplant risk.

Such an expansion has important equity and operational implications. Centers that currently receive few KDPI < 20% offers would gain access to more “acceptable” kidneys, helping to level geographic and center-based disparities in wait-list mortality. Because EDAM incorporates both donor characteristics and immunologic factors, it allows for more precise risk stratification than KDPI alone; this may translate into more nuanced matching and better use of scarce resources. From a systems perspective, directing more organs into the longevity-matching stream could streamline offer chains, reduce organ discards, and decrease cold ischemia times, all of which contribute to greater allocation efficiency.

Implementing an EDAM-based low-risk definition would require updating match-run algorithms and retraining allocation staff; however these logistical challenges are outweighed by the potential gains in patient benefit and system performance. Prospective pilot studies or phased rollouts would help confirm that the expanded low-risk pool continues to perform equivalently in real-world practice. If validated, integrating EDAM into the Kidney Allocation System represents a relatively straightforward policy adjustment with the capacity to improve fairness, efficiency, and outcomes across kidney transplantation. To recite, EDAM shares core predictors with KDPI but add value in competing-risks target assessment (graft failure cumulative incidence, death as competitor), calibration-first transportability testing (internal-external crossvalidation), and a continuous handling of key donor variables—features well-aligned with the clinical decision at offer acceptance, where recipient mortality should not obscure organ quality assessment.

However, caution is warranted when comparing EDAM to KDPI because the 2 scores target different end points. EDAM is built on the cumulative incidence of graft failure with death as a competing event (organ-survival estimand), whereas KDPI was derived for overall graft failure (includes death). In this manuscript, our comparison is therefore classification-focused only; we evaluated how each score labels “low-risk” kidneys and the resulting cumulative incidence of graft failure within those low-risk groups. Notably, nearly half of kidneys classified as low risk by EDAM but not by KDPI exhibited indistinguishable long-term graft outcomes from the conventional KDPI < 20% group, implying substantial misclassification under KDPI and lost utilization opportunities. These findings support EDAM’s ability to safely broaden the low-risk pool for organ acceptance. Nevertheless, because the outcomes or estimands differ, direct performance comparisons between EDAM and the original KDPI should be interpreted with caution; our head-to-head results pertain to classification and observed organ-survival outcomes, not to equivalence across different end points.

We adopted geography-coherent super-regions to test EDAM under credible domain shifts that resemble real-world variation in donor management, allocation logistics, and recipient case-mix. This improves on random crossvalidation by challenging the model in distinct practice environments while maintaining enough data per fold for precise calibration. Using geography-coherent super-regions allowed us to test EDAM under credible practice variation—differences in donor management, allocation logistics, and case-mix—rather than random splits. This approach challenges the model in settings that resemble how it would actually be deployed, while maintaining enough data per fold for precise calibration. The resulting fold-level slopes and pooled estimate indicate that EDAM’s calibration is robust across distinct care ecosystems, supporting its practical transportability. Future work should focus on geographical validation in comparable health systems and on continuous performance monitoring within UNOS to ensure sustained calibration as donor practices evolve.

Our study remains subject to the inherent limitations of retrospective observational research. Unmeasured donor factors may exist that, if included, could further refine the risk stratification provided by EDAM. Residual confounding remains a concern despite adjustments for recipient and transplant-related factors. Changes in organ acceptance practices or improvements in the management of high-risk organs over time may also affect the applicability of our findings. Specifically, if future practices lead to increased utilization of organs with unmeasured high-risk features, the current EDAM may underestimate the true risk associated with these organs. Moreover, selection bias is possible because of the exclusion of cases with incomplete data. External validation is needed to assess the EDAM score among different populations. Because serial donor creatinine measurements were unavailable, the model relied on terminal creatinine values. Incorporating dynamic trajectories, where available, could further refine discrimination between reversible acute kidney injury and chronic donor dysfunction.

Although the EDAM categories were developed to provide a clinically interpretable framework, a notable limitation is that these specific thresholds, including the primary low-risk cutoff of 0.80, were derived in a data-driven manner within the current UNOS dataset. Although our internal-external cross-validation and bootstrap analyses confirm the robustness of the continuous score and the clear separation of the cumulative incidence functions within the UNOS data, these categorical boundaries have not yet been externally validated in independent international cohorts. Because the clinical application of EDAM will likely rely on these categorical assignments for allocation decisions (such as longevity matching), it is critical to acknowledge that these thresholds may require recalibration when applied to different health care ecosystems with varying donor management practices or patient demographics. Until such external validation is performed, these categories should be viewed as evidence-based tiers within the US transplant system rather than universal boundaries. Future research must focus on validating these specific cutoffs in diverse geographic populations to ensure the model’s categorical performance remains stable and equitable across different clinical contexts.

In summary, EDAM stands out as a novel, donor-focused risk stratification model with clear methodological advantages over KDPI. It reliably performs across varied recipient demographics—age and ethnicity—highlighting its potential to enhance transplant decision-making. By integrating EDAM into organ allocation, transplant centers may improve the precision and fairness of kidney distribution, potentially reducing wait times and optimizing graft survival rates.

## Disclosure

TF is a current employee of the United States Veterans Health Administration. All the other authors declared no competing interests.

## Supplementary Material

Supplementary File (PDF) **Table S1.** Adjusted effects of candidate donor predictors prior to EDAM variable selection. **Table S2.** Comparison of donor-only versus adjusted models for graft failure.

## Footnotes

Supplementary File (PDF)
**Table S1.** Adjusted effects of candidate donor predictors prior to EDAM variable selection. **Table S2.** Comparison of donor-only versus adjusted models for graft failure.
