# Supplementary material for: Equitable Donor Assessment Model of Deceased Donor Kidney Quality
Source: Kidney Int Rep. 2026 Jan 21;11(4):103790. doi: 10.1016/j.ekir.2026.103790 (PMC12950383; doi:10.1016/j.ekir.2026.103790)
Supplement: Supplementary File (PDF) — Table S1. Adjusted effects of candidate donor predictors prior to EDAM variable selection. Table S2. Comparison of donor-only versus adjusted models for graft failure. [file mmc1.pdf]

Predictor	Category / Contrast	SHR (95% CI)	p-value
**Donor sex**	Male vs female	0.931 (0.895 – 0.968)	< 0.001
**Donor age**	Per year increase	1.0127 (1.011 – 1.014)	< 0.001
**Cause of death**	Stroke vs anoxia	1.133 (1.080 – 1.190)	< 0.001
	Trauma vs anoxia	0.973 (0.929 – 1.020)	0.261
	Tumor vs anoxia	1.250 (0.966 – 1.619)	0.090
**Donor BMI**	Per kg/m² increase	0.993 (0.990 – 0.996)	< 0.001
**Donor CMV serology**	Positive vs negative	1.072 (1.031 – 1.115)	< 0.001
**History of hypertension**	0–5 years vs none	1.177 (1.113 – 1.245)	< 0.001
	6–10 years vs none	1.190 (1.095 – 1.292)	< 0.001
	> 10 years vs none	1.343 (1.241 – 1.452)	< 0.001
	Duration unknown vs none	1.189 (1.084 – 1.305)	< 0.001
**History of diabetes**	0–5 years vs none	1.250 (1.141 – 1.370)	< 0.001
	6–10 years vs none	1.612 (1.418 – 1.832)	< 0.001
	> 10 years vs none	1.931 (1.724 – 2.163)	< 0.001
	Duration unknown vs none	1.168 (1.005 – 1.357)	0.042
**Donor creatinine**	Per mg/dL increase	1.024 (1.006 – 1.042)	0.008
**Proteinuria in urine**	Unknown vs no	0.927 (0.731 – 1.175)	0.530

	Yes vs no	1.042 (1.004 – 1.082)	0.031
**Donation after circulatory death (DCD)**	Yes vs DBD	1.040 (0.990 – 1.093)	0.115

Supplementary Table S1. Adjusted effects of candidate donor predictors prior to EDAM variable selection

Results are derived from the generalized linear model (clog–log link) for death-censored graft failure, adjusted for recipient and transplant covariates. The table shows subdistribution hazard ratios (SHR), 95% confidence intervals (CI), and p-values for all candidate donor factors evaluated prior to variable selection.

Predictor	Category / Comparison	Unadjusted SHR (95% CI)	p-value	Adjusted SHR (95% CI)	p-value
Donor sex	Male vs female	0.935 (0.901–0.970)	<0.001	0.934 (0.898–0.971)	0.001
Donor age	Per year increase	1.005 (1.003–1.006)	<0.001	1.013 (1.011–1.014)	<0.001
Cause of death	Stroke vs anoxia	1.158 (1.107–1.212)	<0.001	1.125 (1.073–1.181)	<0.001
	Trauma vs anoxia	1.013 (0.969–1.059)	0.566	0.970 (0.926–1.017)	0.203
	Tumour vs anoxia	1.181 (0.915–1.524)	0.202	1.240 (0.958–1.606)	0.103
Donor BMI	Per kg/m² increase	0.995 (0.992–0.997)	<0.001	0.993 (0.990–0.996)	<0.001
Donor CMV serology	Positive vs negative	1.087 (1.048–1.128)	<0.001	1.072 (1.031–1.114)	<0.001
History of hypertension	0–5 years vs none	1.159 (1.100–1.222)	<0.001	1.178 (1.114–1.246)	<0.001
	6–10 years vs none	1.167 (1.080–1.261)	<0.001	1.187 (1.093–1.289)	<0.001
	>10 years vs none	1.284 (1.192–1.383)	<0.001	1.343 (1.242–1.452)	<0.001
	Duration unknown vs none	1.156 (1.058–1.264)	0.001	1.191 (1.086–1.307)	<0.001
History of diabetes	0–5 years vs none	1.231 (1.129–1.343)	<0.001	1.247 (1.138–1.367)	<0.001
	6–10 years vs none	1.524 (1.348–1.724)	<0.001	1.607 (1.414–1.827)	<0.001
	>10 years vs none	1.852 (1.665–2.061)	<0.001	1.915 (1.709–2.145)	<0.001
	Duration unknown vs none	1.189 (1.033–1.369)	0.016	1.180 (1.017–1.370)	0.029
Donor serum creatinine	Per mg/dL increase	1.000 (0.984–1.017)	0.992	1.022 (1.005–1.040)	0.013
Proteinuria	Unknown vs no	1.000 (0.803–1.244)	0.998	0.944 (0.745–1.195)	0.632

	Yes vs no	1.050 (1.013–1.088)	0.007	1.045 (1.006–1.085)	0.022

Supplementary Table S2. Comparison of donor-only versus adjusted models for graft failure.

Comparison of donor-only and adjusted multivariable models for death-censored graft failure. The adjusted model includes transplant and recipient factors to account for confounding and isolate the independent effect of donor attributes. Adjustment led to minimal attenuation of most coefficients, confirming robustness of the donor-driven risk associations used in the final EDAM score.
